# An Evo-Devo Approach to Thyroid Hormones in Cerebral and Cerebellar Cortical Development: Etiological Implications for Autism

**DOI:** 10.3389/fendo.2014.00146

**Published:** 2014-09-09

**Authors:** Pere Berbel, Daniela Navarro, Gustavo C. Román

**Affiliations:** ^1^Departamento de Histología y Anatomía, Facultad de Medicina, Universidad Miguel Hernández, Alicante, Spain; ^2^Department of Neurology, Weill Cornell Medical College, Cornell University, New York, NY, USA; ^3^Methodist Neurological Institute, Houston, TX, USA

**Keywords:** evo-devo, cortical development, autism, thyroid hormones, hypothyroidism

## Abstract

The morphological alterations of cortical lamination observed in mouse models of developmental hypothyroidism prompted the recognition that these experimental changes resembled the brain lesions of children with autism; this led to recent studies showing that maternal thyroid hormone deficiency increases fourfold the risk of autism spectrum disorders (ASD), offering for the first time the possibility of prevention of some forms of ASD. For ethical reasons, the role of thyroid hormones on brain development is currently studied using animal models, usually mice and rats. Although mammals have in common many basic developmental principles regulating brain development, as well as fundamental basic mechanisms that are controlled by similar metabolic pathway activated genes, there are also important differences. For instance, the rodent cerebral cortex is basically a primary cortex, whereas the primary sensory areas in humans account for a very small surface in the cerebral cortex when compared to the associative and frontal areas that are more extensive. Associative and frontal areas in humans are involved in many neurological disorders, including ASD, attention deficit-hyperactive disorder, and dyslexia, among others. Therefore, an evo-devo approach to neocortical evolution among species is fundamental to understand not only the role of thyroid hormones and environmental thyroid disruptors on evolution, development, and organization of the cerebral cortex in mammals but also their role in neurological diseases associated to thyroid dysfunction.

## Introduction

Evolutionary developmental biology (evo-devo) studies the developmental processes of different organisms to determine the ancestral relationships between them and to discover how developmental processes evolved. It addresses the origin and evolution of embryonic development and the modifications of developmental process that produced novel features (Wikipedia, accessed August 2014). Evo-devo teaches us that some fundamental developmental processes are preserved by the evolution among species (1). The evo-devo approach is not only becoming crucial for the modern study of evolution but also it helps in the understanding of morphofunctional alterations in human psychiatric diseases. For instance, autism spectrum disorders (ASD) show abnormal function of cortical areas, such as the frontal or associative neocortices that are minimally present in rodents (2, 3). An approach to the etiologic factors of psychiatric diseases can be inferred by the study of homologous genetic pathways that lead to similar developmental processes in both humans and other mammals. A second issue is that several psychiatric diseases, including ASD, show a wide spectrum of different phenotypes, which are the result of both genetic (nature) and environmental (nurture) factors (4); including among the latter the interaction of comorbid disorders such as hypothyroidism and hypothyroxinemia (5). We begin this review with a summary of thyroid hormone synthesis, transport, and cell actions, which are regulated by a very complex assembly of transporters, deiodinases, receptors, and cofactors. As such, tissues have some control over thyroid hormone action, independent of circulating levels of thyroid hormones. We continue with the analysis of the role of thyroid hormones at different phases of brain development and maturation, focusing our attention on vulnerable periods. These periods occur during gestation and lactation when genetic and environmental factors, which include nutrients and chemical contaminants, interfere with maternal and offspring thyroid health. There is evidence that anatomical characteristics of autistic brains represent defects in processes that occur early in development, in the first half of gestation. Moreover, genomic studies have revealed a catalog of critical genes for these processes that are regulated by thyroid hormones. Finally, recent studies have reported that thyroid hormone deficiency might contribute to increase the number of autism phenotypes, and that disorders associated with hypothyroidism and hypothyroxinemia, such as intellectual impairment, seizures, and anxiety, are comorbid of ASD.

## Thyroid Function during Brain Development

Thyroid hormones (T4, thyroxine; and T3, 3,5,3′-triiodo-l-thyronine) are synthesized in the thyroid gland and are transported to different tissues and organs where they regulate growth, maturation, and function in many organs and systems of vertebrates. In particular, the mammalian central nervous system (CNS) is an important target of thyroid hormones from fetus to adult. However, the maximal vulnerability of the CNS to thyroid hormone imbalance occurs during the earliest stages of brain development (6–15).

In target cells, thyroid hormones can exert their action at three levels: nuclear and mitochondrial (genomic) and non-genomic (16). Genomic actions include (1) thyroid hormone cell membrane transport, (2) thyroid hormone metabolism (involving its activation/degradation), and (3) binding to nuclear thyroid hormone receptors (TRs, also known as THRs), which are ligand-regulated transcription factors (17–25).

### Thyroid hormone cell membrane transport

Thyroid hormone cell membrane transport is mediated by four families of transporters: the Na^+^/taurocholate cotransporting polypeptide (NTCP), the organic anion transporting polypeptide (OATP), monocarboxylate transporter (MCT), and the heterodimeric amino acid transporter (HAT) (26). From these, Oatp14, Mct8, Mct10, Lat1, and Lat2 have been found to be expressed in the brain (20, 26–33).

### Thyroid hormone metabolism (activation/degradation)

Three selenoproteins catalyzing the deiodination of T4 (thyroxine) and T3 (the active form for the genomic action) have been identified: type 1 (D1), type 2 (D2), and type 3 (D3) iodothyronine deiodinases. Only D2 and D3 have been found expressed in the CNS. D2 has been found in the astrocytes and tanycytes [special ependymal cells, Ref. (34)] and mediates the local generation of T3. D3 mediates the degradation of T3 to T2 (diiodothyronine, 3,5-diiodo-l-thyronine) and T4 to rT3 (35–37). In addition to deiodination, iodothyronines are also metabolized by conjugation of the phenolic hydroxyl group with sulfate or glucuronic acid (38).

### Thyroid hormone nuclear receptors

In the CNS, there are three nuclear TR isoforms with high-affinity to T3: TRα1 (codified by the *THRA* gene), TRβ1, and TRβ2 (codified by the *THRB* gene) (17, 20, 39, 40). TRα1 is the most ubiquitous; it has been detected in the rat brain by embryonic day 12 (E12) and in the human brain by the 10th week of gestation (41–43), regulating the expression of genes involved in the development and maturation of the brain (44), while TRβ1 is mostly expressed in the adult. In addition, N-terminal truncated TRα1 (also known as p43) can serve as a T3-dependent transcription factor that initiates global mitochondrial transcription (16, 45, 46).

Recent studies have shown that thyroid hormone signaling is more diverse and complex than initially concluded. For instance, apart from the canonic role of thyroid hormones mentioned above, novel THRs synthetic ligands might also modulate TRs action, and intra and extracellular signals can affect cell sensitivity to T3 influencing *TRs* gene expression, TRs translation and its transport into the nucleus, and the recruitment of co-activators/-inhibitors (21, 24, 47). Furthermore, thyroid hormones can show non-genomic actions by binding to cell surface or cytoplasmic receptors and by interacting with other signaling pathways (16, 21, 48).

In rodents and humans, almost all T3 found in the fetal cerebral cortex is generated through local deiodination of circulating maternal T4 (13, 49, 50). The fetal dependence on maternal T4 is due (i) to the late development of the fetal thyroid gland (in rodents thyroid function begins by E17–18 and in humans by the 18–20 gestational week) and (ii) to the increased activity of D2 and D3 deiodinases in placenta and fetal tissues (13, 35, 51, 52). As a consequence of the increased activity of deiodinases in the fetus, serum T3 levels are maintained low and the local generation of cerebral T3 from T4 is enhanced (13, 50). To respond to this requirement, there is an estrogen-dependent increase of maternal thyroid function that transiently induces an increase of (i) circulating thyroxine-binding globulin, affecting the T4 extra-thyroidal pool, and of (ii) human chorionic gonadotropin, transiently stimulating thyrocytes (53). This increased maternal thyroid function consequently needs increased iodine intake.

## Nutritional and Environmental Factors Affecting Thyroid Function

Several factors can affect thyroid function during gestation and early postnatal development, including genetic mutations, infections, nutrients, and environmental contaminants. Iodine deficiency from inadequate alimentary habits is the most common cause of maternal and fetal thyroid dysfunction (54–57). In addition, selenium (a component of deiodinases), iron (a component of the prosthetic heme group associated to the thyroperoxidase), and other micronutrients are required for an adequate life-long thyroid function, especially during development and adolescence (58). Moreover, environmental anti-thyroid contaminants are acquiring increased importance (55, 59–67).

## Thyroid Function-Disrupting Chemicals from Environmental Contaminants

A thyroid function-disrupting chemical is an exogenous chemical, or mixture of chemicals, that can interfere with any aspect of hormone action (67). The mechanisms of action of disrupting chemicals on thyroid function are not fully understood; some may reduce serum T4 without increasing serum TSH while others may interfere with thyroid hormone action at sites other than the thyroid gland without altering serum TSH levels (21, 67). Howdeshell (59) listed synthetic chemicals that interfere with thyroid hormone synthesis, transport, and metabolism. Some are quite specific such as perchlorate salts that block the sodium/iodide symporter (68), but the majority affects several phases of thyroid hormone action. Some thyroid disruptors are consumed in the diet (5, 63); for instance, plant isoflavonoids such as genistein and daidzein from soy inhibit thyroperoxidase that catalyzes iodination and thyroid hormone biosynthesis; thiocyanate from cassava not only blocks iodine uptake by thyroid and mammary glands but also interferes with thyroid peroxidase. Organochlorides (including mostly DDT and its derivative: *p*,*p*′-DDE, dichlorodiphenyl dichloroethylene; HCB, hexachlorobenzene; PBB, polybrominated biphenyls; and PCB, polychlorinated biphenyls) interfere with thyroid function acting upon iodine uptake, thyroid peroxidase action, thyroid hormone binding proteins, and thyroid hormone metabolism, resulting in a wide spectrum of thyroid-related syndromes (59, 69). The increased use of nanoparticles in several industrial, consumer, and medical applications has revealed their unique physico-chemical properties. However, *in vitro* and *in vivo* studies have shown that they may have toxic effects on the endocrine system (70). It has been found that Ag-nanoparticles and cadmium telluride-quantum dots alone induced a reduction in the expression TRβ (71).

## Iodine Deficiency Disorders and Neurodevelopmental Damage

As mentioned before, during gestation, the mother must produce sufficient amounts of thyroid hormones (fundamentally T4) for herself and her fetus. Iodine intake is the principal source of circulating inorganic iodine; therefore, sufficient iodine is critical for the thyroid gland to produce adequate amounts of thyroid hormones (9, 13, 53, 57, 72–77). The fetus also depends on the mother for its iodine supply, as does the neonatal thyroid during lactation (78). To achieve this, expecting mothers need to double the recommended normal daily intake of iodine for non-pregnant women by 250–300 μg/day (79).

Useful food strategies developed to increase iodine intake in iodine-deficient areas include (i) use of iodinated salt in the household, (ii) incorporation of iodine to industrially elaborated foods (i.e., bread, milk, and cheese), and (iii) dietary diversification (i.e., consuming food from iodine-sufficient areas and seafood). Despite these strategies, inadequate iodine intake actually affects a large number of women during pregnancy and lactation, and this situation currently persists even in countries classified as free of iodine deficiency where iodized salt consumption has been promoted for years (79–84).

Iodine deficiency is one of the most frequent causes worldwide of preventable mental retardation in children (85). A wide spectrum of iodine deficiency disorders has been described during gestation and the early postnatal period (<3 years of age), ranging from abortion, stillbirths, congenital anomalies, deafness, cretinism, neurocognitive delay, epilepsy, schizophrenia, ASD, as well as attention deficit hyperactive disorder (ADHD), among others (3, 8, 63, 72, 86–94). In children, the severity of the neurodevelopmental damage caused by iodine deficiency during gestation depends on several factors: (i) the developmental period affected, (ii) its severity, (iii) the deficiency of other nutrients such as selenium and iron, and (iv) the interaction with thyroid function disruptors (9, 10, 14, 57, 58). Epidemiological studies performed in several countries have shown that hypothyroxinemia due to mild iodine deficiency during gestation causes neurological alterations, including low IQ in children (8, 87, 88, 95–99). As mentioned above, iodine deficiency in conjunction to the deficiency of other nutrients and the interaction with thyroid function disruptors will cause a wide spectrum of syndromes associated to thyroid pathologies. In countries with severe iodine and selenium deficiencies, a high incidence of Kashin–Beck osteoarthropathy associated with cretinism has been observed (100). The incidence of myxedematous cretinism increases in countries where severe iodine and selenium deficiency is associated with high intake of thyroid disruptors found in foodstuffs such as cassava, which contain thiocyanate (5, 101). The study of the alterations resulting from nutritional deficiencies in combination with thyroid function disruptors should contribute to our understanding of the multiple syndromes observed in thyroid diseases.

## Critical Issues of Cerebral Cortex Development

For ethical reasons, the role of thyroid hormones on brain development is currently studied using animal models, usually mice and rats. However, although there are basic common developmental principles regulating brain development between mammals, there are also important differences. For instance, the understanding of how different types of neocortex evolved depends on determining not only the numbers and types of cortical areas that exist but also how the internal organization of those areas was modified in the various lines of evolution, including modifications in columnar organization (102). Sexual dimorphism among species also plays an important role, particularly in humans, while in rodents little is known about sex differences between cerebral hemispheres (103). Changes in the organization and size of neocortex also are reflected in the size of cortico-cortical and subcortical projections, in turn affecting target areas (104). Thus, increasing our evo-devo knowledge on neocortical evolution among species (2) will help us to understand not only the role of thyroid hormones and environmental thyroid disruptors on the development, organization, and evolution of the cerebral cortex in mammals (105), but also their role in human associated diseases. The evo-devo considers crucial for the evolution that homologous developmental gene networks are shared among species (1), and it emerges from the relationship between developmental biology and evolution, which in turn are dynamically coupled (106). For instance, basic gene networks involved in symmetric divisions of ventricular neuroblasts during cerebral corticogenesis are common in rodents and humans, while humans evolved by increasing the number of symmetrical divisions, which results in an increased number of cortical columns and therefore an increased cortical surface [see figure 2A by Rakic (2)]. Apart from homology, convergence can also bring solutions for common functional problems. However, little is known on how functional needs have selected different functional networks to generate a similar function between different species, such as the wings of birds and bats (1). Genetic (nature) and environmental (nurture) factors cooperate along time, resulting in differentiation (4). Psychiatric diseases, such as ASD, occur in cerebral areas (e.g., frontal and associative) that are not present in rodents; however, many homologous functional networks, like those involved in radial migration, have been preserved. Furthermore, ASD show a wide spectrum of different phenotypes, resulting in different degrees of morphofunctional alterations and in the concurrence of different comorbid disorders (107). Thyroid hormone deficiency increases comorbidity and the risk of developing ASD (3). For instance, thyroid hormone deficiency during neocorticogenesis results in abnormal development of cortical gamma-aminobutyric acid (GABA)-ergic neurons, which cause altered columnar function in the cerebral cortex and ASD comorbid seizures (108).

The cerebral cortex in all mammalian species, including humans, differs from the development of other organs of the body and even from the rest of the brain. It is a three-dimensional sheet of layers, parallel to the pial surface, mostly composed of projection (or glutamatergic pyramidal) and local neuronal circuits (or glutamatergic and GABAergic interneurons) organized in vertical (or radial) columns that are stereotypically interconnected and share extrinsic connectivity in order to achieve their functions (109). During telencephalic corticogenesis in mammals, including humans, layer I and subplate (deriving from the superficial primordial plexiform layer) are the first cortical layers to appear (110). Subsequently, young cortical neurons begin to migrate radially from the ventricular zone into the superficial cortical plate, adjacent to layer I, following an “inside-out” gradient (111). While in rodents, the neurogenesis of layer I is arrested when radial migration begins, in primates neurogenesis continues during all the periods of corticogenesis (112). Neurons migrate radially to the increasingly distant cortex following the scaffolding of a transient population of radial glial cells (113), in which many signaling pathways – such as reelin, metabolic functions, and gene expression must be involved (114). This phase of corticogenesis is of capital importance because an evo-devo approach of neocortical development and evolution can be explained by the radial unit hypothesis proposed by Rakic (115). As reported by Rakic (2) and mentioned above, increased number of symmetrical divisions will increase the number of functional columns, resulting in increased tangential cortical surface, while that of asymmetrical ones will increase the number of cells per column, resulting in increased cortical thickness [see also figure 2A by Rakic (2)]. The final number of these divisions will depend of apoptotic, anti-apoptotic, or inhibitory factors, and will give rise to either the small lissencephalic cerebrum of rodents or to the larger convoluted cerebrum of humans, as well as to the emergence of new functional areas, such as the prefrontal cortex and associative perisylvian areas (2). The graded expression of transcription factors such as Emx2, Pax6, Coup-Tf1, and Sp8 are implicated in the arealization of the neocortex (116, 117). Deletion or overexpression of these factors results in changes in gene expression, contractions, and expansions in the sizes of cortical fields, and altered patterns of connectivity from the dorsal thalamus (117). Emx2 and Fgf genes share reciprocal functions in regulating cortical patterning; in the frontal cortex, this is accomplished at least in part through controlling the levels of Erm, Er81, Pea3, and Sp8 expression (118, 119). These results support the protomap model (115, 120, 121) because neurons are committed to their areal position at the time of their last cell division (the asymmetrical one) in the proliferative zones in the absence of thalamic afferent inputs, although individual cortical areas may be selectively changed in size during the course of evolution by altered expression signals of their downstream transcription factor signaling mechanisms, as mentioned above (2). In addition, changes in gene expression extrinsic to the neocortex in response to physical stimuli in a particular environmental context might play a crucial role in the formation of domains and areas in the neocortex (117, 122, 123).

In rodents, radially migrating neurons comprise about 80% of the total cortical neurons and will become glutamatergic neurons. The remaining 20% of the cortical neurons migrate tangentially (i.e., parallel to the pial surface) from the ganglionic eminences to their target area and will become local circuit neurons, mostly GABAergic neurons (124–126). In humans, differently from rodents, a subset of neocortical GABAergic neurons [Mash1-positive; a marker for precursors of glutamic acid decarboxylase (GAD)-expressing cells] originates in the ventricular/subventricular zones of the dorsal telencephalon as a distinct neuronal stem cell lineage [Ref. (127, 128); see figure 5 by Rakic (2)]. The identification of the telencephalic origin of local circuit neurons in cerebral cortex of mammals is of capital importance to understand mechanisms operating during primate brain evolution (2, 129, 130) and the pathogenesis of congenital and acquired neurological disorders, such as ASD, related to defects of separate classes of local circuit neurons (131, 132).

**Figure 1:**
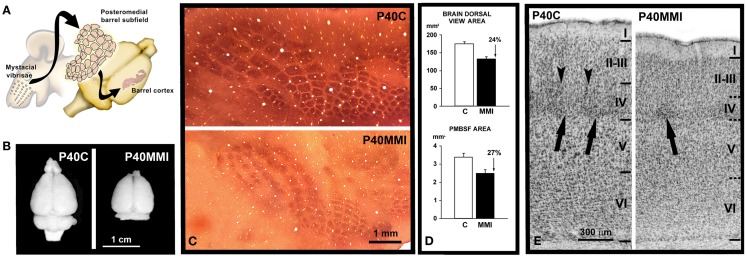
**Reduced development of cortical maps in developmental hypothyroidism**. **(A)** Cartoon showing the posteromedial barrel subfield of the primary somatosensory cortex in the brain of a rat. Note the correspondence between mysticial vibrises and the barrels of the posteromedial barrel subfield. **(B)** Brain dorsal views at P40 of control (C) and MMI pups. **(C)** Computer reconstruction from photomicrographs of serial tangential sections through layer IV, showing cytochrome oxidase labeling in the barrel cortex of normal and hypothyroid rats. Note the reduced tangential extension of the cytochrome oxidase labeling in hypothyroid with respect to normal rats. **(D)** Area measurements in normal and hypothyroid rats. The dorsal view brain area was, on average, 24% smaller in hypothyroid rats (upper). A similar reduction (on average, 27%) was observed in the PMBSF tangential area (lower). **(E)** Photomicrographs of cresyl violet stained coronal sections showing the cytoarchitecture of the barrel cortex of the primary somatosensory cortex at P40 in control (C) and transient MMI treated pups (MMI treatment begun at E12 and finished at E15). Borders between layers (horizontal lines) are clear-cut in C whereas they are more blurred in MMI12 pups. In layer IV of C and dMMI pups, barrels (arrow) are normal and well-defined and demarcated by septae (arrowheads). In contrast, barrels in layer IV of MMI1 pups are not seen. In developmentally hypothyroid pups there is a 10–15% reduction in the cortical thickness of MMI pups compared to controls. **(A)** Modified from Berbel and Morreale de Escobar (57). **(C,D)** Modified from Berbel et al. (192). **(E)** Modified from Ausó et al. (158).

**Figure 2:**
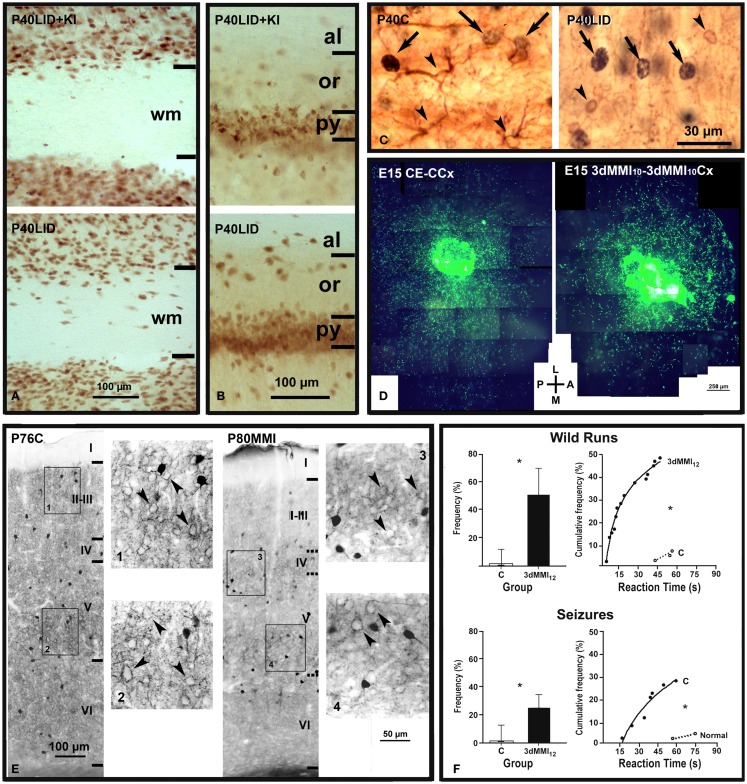
**Abnormal neuronal radial and tangential migration, inhibitory local circuits and increased audiogenic seizures in developmental hypothyroidism and hypothyroxinemia**. **(A,B)** Photomicrographs of NeuN-immunostained coronal sections of the primary somatosensory cortex **(A)** and hippocampal CA1 **(B)** in LID + KI (rats fed low iodine diet plus approximately 10 μg iodine per day, during gestation and postnatally) and LID (rats fed low iodine diet) progeny at P40. The number of NeuN-labeled neurons increases both in subcortical white matter [wm **(A)**] and in strata oriens (or) and alveus [al **(B)**] of hippocampal CA1 of LID pups as compared with LID + KI pups. **(C)** GFAP- and CNP-positive astrocytes (left panel) and oligodendrocytes (right panel), respectively (arrowheads), and BrdU-positive nuclei (arrows) are shown in layer V of C and LID pups. LID rats received single BrdU injections at E14, E15, and E16. Note that both GFAP-positive astrocytes and CNP-positive oligodendrocytes are BrdU-negative. **(D)** Low power fluorescent photo- micrograph collage illustrating the tangential distribution of GFP-MGE control migrating neurons (control explant, CE) in wild control flat cortical mounts at E15 (control cortex, CCx; left), and GFP-MGE hypothyroxinemic migrating neurons (3dMMI_10_) in hypothyroxinemic flat cortical mounts (3dMMI_10_Cx; right). Note that migrating neurons toward the medial (M) region in the hypothyroxinemic cortical mount (right) expand less than those migrating in the control cortical mount (left). 3dMMI_10_ rats received MMI treatment from E10 to E12. **(E)** Photomicrographs through layer V of the auditory cortex immunostained for parvalbumin in normal (C) and hypothyroid (MMI treatment from E14 onward) rats. In normal rats, immunoreactive cells, processes and perisomatic puncta can be seen. In MMI rats, immunoreactive cells, processes and perisomatic puncta can also be seen but they are less prominent than in normal rats. **(F)** Responses of C and 3dMMI12 (MMI treatment from E12 to E15) pups to an acoustic stimulus. Histograms on the left correspond to the proportion (median with 25th and 75th percentiles) of pups responding with wild runs and with wild runs followed by a seizure, respectively. Graphs on the right represent the cumulative frequency of pups from the same groups that respond with wild runs alone or followed by a seizure, respectively, at the intervals after onset of the stimulus that are shown in the abscissa. (*) Indicates a statistically significant difference compared with control. **(A–C)** Modified from Lavado-Autric et al. (164). **(D)** Modified from Cuevas et al. (159). **(E)** Modified from Berbel et al. (211). **(F)** Modified from Ausó et al. (158).

In rats, the bulk of neocortical radial migration starts by embryonic day 13 (E13), while the last cohort of cells leaves the ventricular zone by E20 (133). During this process of radial and horizontal migrations, the subplate neurons attract “waiting” afferents from ipsilateral and contralateral cortical areas (including associative and commissural connections), and subcortical connections [including thalamic, nucleus basalis, and monoamine connections (2), see also the figure 2B of this reference]. At the end of this process, neurons and glial cells grow and differentiate, including the loss of juvenile transient connections, to express their mature phenotype, which also contributes to the radial and tangential expansion of the cortex (134). In humans, neocortical development occurs between the 6th and 24th week of gestation (110, 135). The main waves of radial migration in the human neocortex occur during the first half of gestation, with peaks at 11 and 14 weeks of gestational age (110, 135), and mostly before onset of fetal thyroid hormone secretion by the 18th week of gestation (136). This roughly corresponds to waves of cell migration studied in rats (10), which also occur before onset of fetal thyroid hormone secretion, by E17.5–18 (136). Despite the longer development and maturation of the CNS in humans compared with rats, similarities may be established when the onset of fetal thyroid gland secretion is taken as the reference point. However, when comparing the rodent lissencephalic and the primate convoluted mature neocortex, the major differences are found in the tangential rather than in the radial expansion [see figure 1 by Rakic (2)].

## Experimental Models to Study Cortical Alterations Caused by Thyroid Hormone Deficiency

Several experimental models have been developed to study alterations in the CNS caused by thyroid hormone deficiency. These models can be grouped into (i) genetic mutants, (ii) surgically induced hypothyroidism, (iii) metabolite deficient diets, and (iv) thyroid function disruptor models.

Several genetic models were developed during the last decades to study different forms of developmental and postnatal hypothyroidism, such as congenital hypothyroidism (137). Genetic models can be classified into two main groups: (1) mutations affecting thyroid gland development and function, and (2) mutations affecting thyroid hormone sensitivity, which includes thyroid hormone cell membrane transport, metabolism, and action (25). The first group includes mutations of the TSH receptor (*hyt*^−/−^ mice) (138) and agenesis or functional impairment of thyrocytes (*TTF1*^−/−^, *TTF2*^−/−^, and *Pax8*^−/−^ mice) (139). The second group includes thyroid hormone transporters mutants such as *Mct8*^−/y^ (140, 141), *Mct8*^−/−^ (142), and *Lat2*^−/−^ (143). These mutant mice have provided new data to understand thyroid hormone transport in the cell membrane and clarified the physiopathology of the Allan–Herndon–Dudley syndrome, which is caused by MCT8 defect (141, 144–146). Thyroid hormone metabolism in the brain has been studied using different mutant mice affecting D2 and D3 expression (*Dio2*^−/−^, Dio3^−/−^, and *Dio2*^−/−^*Mct8*^−/y^ mice) (147–149). Important genes associated to cortical development are affected in Dio mutants. In particular, the neuronal genes *Gls2* (glutaminase 2), *Nefh* and *Nefm* (heavy and medium neurofilament polypeptide), *Sema7a* (semaphorin 7A), *Shh* (sonic hedgehog), *Col6a1* and *Col6a2* (type VI α1 and α2 collagen), as well as *Slc1a3* (glial high-affinity glutamate transporter) and Itga7 (integrin α7), among others, found in glial cells (148). Mutations of the *TR* gene include *TR*α^−/−^, *TR*β^−/−^, and *TR*α^−/−^β^−/−^mice, as well as *TR*α and *TR*β knock-in mutations (23, 150). Mutations of *TR*β gene are associated to the Refetoff syndrome (151, 152). A classification of these mutations and their associated syndromes of impaired sensitivity to thyroid hormone has been recently published (25).

The most common models are based on the administration of anti-thyroid drugs interfering either with the thyrocytes iodine uptake by inhibiting the sodium/iodine symporter (e.g., potassium perchlorate and thiocyanate) or with the iodination of thyroglobulin by thionamide and thiourylene drugs such as propylthiouracil (PTU) and methimazole (MMI) (153–155). In addition, PTU (and less MMI) partially inhibits iodothyronine deiodinases affecting the peripheral deiodination of T4 (154, 156). Anti-thyroid treatments result in maternal, fetal, and neonate hypothyroidism of greater or lesser severity (157). MMI treatment was also used experimentally to induce mild and transient maternal hypothyroxinemia at the onset (E12) of fetal neocorticogenesis (158, 159). Models for iodine deficiency during gestation include monkeys (160), sheep (161), and rats (162, 163). These studies have shown changes in the cerebellum with reduction in weight and cell number, and delayed maturation. The influence of iodine deficiency on neocortical development has been studied in rats that are fed a low iodine diet during pregnancy (163–166).

Alternatively, surgical thyroidectomy can be used to induce hypothyroidism (167, 168), when performed in pregnant dams it causes maternal but neither fetal nor neonate hypothyroidism. Recently, late maternal hypothyroidism (LMH) during gestation has been used as a model to study the role of maternal thyroid hormones from the onset of fetal thyroid function (169).

## Alterations in Cortical Development Caused by Thyroid Hormone Deficiencies

### Genes regulated by thyroid hormones involved in brain development

Fundamental genes involved in brain development are regulated by thyroid hormones. The irreversibility and importance of damage will depend on when, where, and how the alterations of gene expression occur (10, 20). Early studies showed that maternal thyroid hormones regulate gene expression in fetal development modulating the expression of *NSP* and *Oct-1* genes; T4 injections produced rapid, transient, and selective effects on gene expression in the fetal brain (170). Additional genes regulated by maternal thyroid hormones included *Nrgn* (neurogranin, also known as RC3), found to be significantly decreased (171), as well as reelin, apolipoprotein E receptor 2 (ApoER2; a reelin receptor involved in the migration young neocortical neurons), very-low-density lipoprotein receptor (VLDLR; a reelin receptor that mediates the stop signal), integrin genes, and genes involved in the downstream phosphorylation of Dab1 (very-low-density lipoprotein receptor 1) (172, 173). cDNA microarray studies have shown a number of genes to be transcriptionally or functionally modulated by T3; most of these are involved in cell division, migration, growth, connectivity, and function of neural cells. Using rat pituitary GC cell line, Miller et al. (174) showed that 358 out of 4,400 genes were regulated by T3; and, in a recent study, Morte et al. (44) found 552 out of 14,209 genes regulated by fetal and maternal thyroid hormones at the end of gestation in rats. The function of some of these genes is unknown but most of them are involved in the regulation of key pathways for the development of the cerebral cortex in rodents and humans. Tables 1–6 list some of the most relevant T3-regulated genes at the transcriptional level. Among those of relevant importance for the development of cortical connections are *Nefh*, *Nefl*, and *Nefm* (coding neurofilament proteins); *Slit1*, *Slit2*, *Nos1*, *Camk4*, and *Creb1* (involved in bifurcation and growth of neural processes); *Sema3B*, *Slit1*, and *Slit2* (guiding axons); and *Slc17a7* (coding vesicular glutamate transporter 1; VGluT1). T3 action on the regulation of the Camk4/Creb pathway and downstream targets (175) in neurons of the CNS is highly relevant since Camk4 has not been found expressed in glial cells (169, 176, 177). Camk4 is directly induced by T3 at the transcriptional level (44), and phosphorylates Creb. Many of the genes under thyroid hormone control contain Creb binding sites in their promoter region (149). On the other hand, Camk4 regulates the transcriptional activity of the TR, which might be due to direct phosphorylation of co-activators or by changing the equilibrium between the co-activators and the silencing mediator for retinoid and thyroid hormone receptors (SMRT) (178, 179). Camk4/Creb pathway and downstream targets are involved in processes such as neurogenesis, biosynthesis, and assembly of cytoskeleton, cell movement and migration, neurite development and maturation, synaptic plasticity, and neurotransmission (44, 180). In humans, Camk4/Creb pathway is involved in psychiatric disorders (181–183). There is a strong evidence for the action of Camk4/Creb pathway in the expression of *FMR1* gene, encoding fragile X mental retardation protein (FMRP) (184, 185). Lack of FMRP causes fragile X syndrome, which is the most common cause of inherited mental retardation and ASD (186, 187). In addition, brain-derived neurotrophic factor (BDNF)/Erk signaling modulates FMRP function, affecting neuronal proliferation and differentiation in the cerebral cortex [Ref. (188, 189); Table 5].

**Table 1:** **Significant T3-regulated genes at the transcriptional level found in the cerebral cortex of rodents, involved in cell division and differentiation: relationship with ASD**.

Symbol^a^	Protein	Process	Alteration/disease
*ADCYAP1R1*	Adenylate cyclase-activating polypeptide receptor (PAC1)	Signaling pathway	Decreased second messenger
*CASP3*	Caspase 3	Protease	Apoptosis. Alzheimer’s disease
*CCND1*	G1/S-specific cyclin-D1	Interact with tumor suppressor protein Rb	Abnormal cell cycle G1/S transition
*CNN1*	Calporin (actin binding protein; fimbrin type)	Actin associated protein	Abnormal cohesion between parental centrioles
***CREB1***	cAMP-responsive element binding protein 1	Transcription factor	Altered development. ASD
***CREM***	cAMP-responsive element modulator	Transcription factor modulating CREB	Altered development. ASD
***CTNNB1***	β-catenin	Regulates the coordination of cell–cell adhesion and gene transcription	Altered asymmetric cell division, epithelial-to-mesenchymal transition. ASD
***DYRK1A***	Dual specificity tyrosine-phosphorylation- regulated kinase 1A	Nuclear signaling	Abnormal cell proliferation and may be involved in brain development. ASD
***GNB1L***	Guanine nucleotide-binding protein subunit β-like protein 1	Six WD40 repeat-containing protein	Abnormal cell cycle progression. Schizophrenia. ASD
***FLT1***	Vascular endothelial growth factor receptor 1	Protein kinase	Abnormal control of cell proliferation and differentiation. ASD
*HIST1H1T*	Histone H1t	Compaction of chromatin	Abnormal cell cycle and differentiation
*HSD11B2*	Corticosteroid 11-β-dehydrogenase isozyme 2	Hydrolysis of cortisol	Cortisol induction of growth-inhibition and/or pro-apoptosis embryonic development
***MAPK1***	Mitogen-activated protein kinase 1 (ERK2)	CREB1 phosphorylation signaling pathway	ASD
*RGS3*	Regulator of G-protein signaling 3	Ephrin-B signaling pathway	Early cell cycle exit and precocious differentiation

*^a^Bold shows T3-regulated genes that have been found to be abnormally expressed in autistic humans. Other genes found in autistic humans not regulated by T3 at the transcriptional level have not been included*.

### Altered neurogenesis and migration during corticogenesis

Indirect observations based on the cell density estimates and brain size measurements suggested a reduced number of cells in the neocortex of developmentally hypothyroid rats (190). Neural progenitors in the ventricular zone of mouse telencephalon express TRα1, Mct8 transporters, and deiodinases, and maternal hypothyroidism reduces the cell cycle length of these progenitors (191). Since in hypothyroid fetuses the bulk of the neocortical BrdU-labeling occurs between E12 and E19 as in control rats (192), the data by Mohan et al. (191) clearly indicated that the total neuronal progenitor number is reduced in the cerebral cortex. Using ^3^H-thymidine labeling, a significant reduction was observed in cell acquisition in the granular layer of the hippocampal dentate gyrus in postnatal PTU treated pups (193). These authors also observed that the radial migration of newly generated hippocampal granular cells could be arrested and that the decreased number of labeled cells in the granular layer might result from deficient migration rather that decreased mitotic activity. Several genes involved in cell cycle regulation during neurogenesis have been found to be regulated by T3 [Ref. (44); Table 1]. CCND1 (G1/S-specific cyclin-D1) is downregulated by T3, resulting in an abnormal cell cycle progression (194). In addition, T3-regulated regulator of G-protein signaling 3 (RGS3) plays a key role in ephrin-B signaling, controlling cell cycle exit, and differentiation of neural progenitors (195), and dual specificity tyrosine-phosphorylation-regulated kinase 1A (DYRK1A) is involved in the control of cell proliferation in ASD, causing arrested brain growth (196). Other T3-regulated genes such as CNN1 (calporin), which is an actin associated protein, also exerts a control of cell cycle during neurogenesis (197). Recently, it has been found that developmental mild and severe hypothyroxinemia and MMI-induced hypothyroidism alters Shh signaling pathway in the cerebellar granule cell precursors, resulting in downregulation of D1 and D2 cyclins, of E2F1 expression, and in reduced cell proliferation (198). However, it still remains unclear to what extent thyroid hormones affect symmetrical and asymmetrical divisions of neocortical progenitor cells. Studies on the mouse barrel cortex (192) suggest that both symmetrical and asymmetrical divisions are altered in hypothyroid rats, because the tangential area of the posteromedial barrel subfield stained with cytochrome oxidase (resulting from symmetrical divisions) and the thickness of the barrel cortex (resulting from asymmetrical divisions) are reduced by 27 (Figures 1A–D) and 12.5% (Figure 1E), respectively, in hypothyroid rats. Nevertheless, from these data we could argue that most likely the symmetrical divisions are comparatively more affected in hypothyroid rats. The reduced thickness of the cortex in hypothyroid rats could be explained by a reduction of the columnar neuropile more than by a reduction in the cellular components of the columns. T3-regulated *CASP3* (caspase 3) and *CTNNB1* (β-catenin) genes are crucial for cerebral cortex expansion (Table 1). Experimental studies using caspase 3 and 9 KO mice (lacking apoptotic signals) (2, 199) and transgenic mice expressing β-catenin (which increases the number of precursor cells) lead to an abnormally convoluted mouse cortex (200). Caspase 3 pathway is downregulated in the cerebral and cerebellar cortices of hypothyroxinemic and hypothyroid rats (201, 202), while β-catenin is T3-downregulated in rat pituitary cultured cells (174). These data show that thyroid hormone deficiency alters the tangential and radial organization of the cortex and might have contributed in the evolutionary elaboration of radial columns, modulating both cortical surface and thickness.

Altered T3-regulated opioid-binding protein/cell adhesion molecule (OPCML; also known as OBCAM) expression affects radial glia function and its transdifferentiation to astrocytes [Ref. (203); Table 2]. In agreement, impaired maturation of radial glia was observed in the hippocampus of pups born to chronic hypothyroxinemic rats (163) and in the neocortex of developmentally hypothyroid pups (204). Abnormal radial migration in the neocortex of developmentally hypothyroid rats was first described in the auditory cortex by combining BrdU and tracer labeling (205). As a result, the radial positioning of migrating neurons was altered, including abnormally located heterotopic neurons in the subcortical white matter (192, 205) (Figure 2C) and corpus callosum (206). Also, altered neuronal migration in the neocortex and hippocampus has been confirmed in hypothyroxinemic rats (158, 159, 164) (Figures 2A–C). Apart from TRs, other nuclear receptors are involved in radial glia maturation and radial migration in the neocortex such as the liver X receptor β (LXRβ) that also regulates the expression of ApoER2 receptor (207). *LXR*β^−/−^ mice showed altered cortical migration of later-born neurons (208) and delayed transdifferentiation of radial glial cells into astrocytes (209). Interestingly, LXRs bind to the same response element on DNA as TRs and sometimes regulate the same genes (150, 210). In fact, it has been shown recently that TRα compensates for the lack of LXRβ in cortical development, and a reciprocal compensatory action can also be hypothesized (207).

**Table 2:** **Significant T3-regulated genes at the transcriptional level found in the cerebral cortex of rodents, involved in cytoskeleton organization and cell migration: relationship with ASD**.

Symbol^a^	Protein	Process	Alteration/disease
*APOER2*	Apolipoprotein E receptor 2 (Lrp8)	Reelin signaling pathway	Alzheimer, major depressive disorder
*CALR*	Calreticulin	Endoplasmic reticulum calcium-binding protein	Abnormal calcium storage in the hippocampus. Alzheimer’s disease
***CREB1***	cAMP-responsive element binding protein 1	Transcription factor	Altered development. ASD
***CREM***	cAMP-responsive element modulator	Transcription factor modulating CREB	Altered development. ASD
*CTSS*	Cathepsin S	Protease	Abnormal microglial function
***DAB1***	Disabled-1	Reelin signaling pathway	Abnormal migration. Alzheimer’s disease, temporal lobe epilepsy. ASD
*DYNLL1*	Dynein light chain 1, cytoplasmic	Microtubule associated protein	Abnormal intracellular transport and motility
*FMOD*	Fibromodulin	Proteoglycan that sequesters TGF-β into the extracellular matrix	Abnormal regulation of proliferation and differentiation of hippocampal granule neurons
***FN1***	Fibronectin	Extracellular matrix protein	Abnormal cell adhesion, growth, migration, and differentiation. ASD
***GNAS***	G-protein α subunit (Gs-α)	Signaling pathway	ASD. ADHD
*HSPD1*	Chaperonin (HSP60)	Chaperone	Prevent traumatic brain injury
***MAPK1***	Mitogen-activated protein kinase 1 (ERK2)	CREB phosphorylation signaling pathway	ASD
***NEFH***, ***NEFM***, ***NEFL***	Neurofilament protein (heavy, medium, and light)	Intermediate filaments	Abnormal neuronal cytoskeleton. ASD
*NOV*	Nephroblastoma overexpressed	Extracellular matrix protein that binds to integrin receptors	Abnormal cell adhesion, migration, proliferation, differentiation,and survival
*OPCML*	Opioid-binding protein/cell adhesion molecule	Cell adhesion molecule	Abnormal proliferation and growth of cortical astrocytes
***PAFAH1B1***	Platelet-activating factor acetylhydrolase IB subunit α (Lis1)	Interact with dynein and VLDLR	Lissencephaly. ASD
***RELN***	Reelin	Extracellular matrix protein	Abnormal migration. Alzheimer’s disease, temporal lobe epilepsy. ASD
***SERPINH1***	Heat shock protein 47	Chaperone	Abnormal collagen binding. ASD
*SLIT1*, *SLIT2*	Slit homolog 1 and 2 proteins	Extracellular matrix protein. Chemorepulsive signal	Abnormal axon guidance. Abnormal angiogenesis
*TGFB2*	Transforming growth factor-β 2	Extracellular matrix protein	Abnormal regulation of proliferation and differentiation of hippocampal granule neurons
*TPM1*	Tropomyosin α-1 chain	Actin associated protein	Abnormal neuronal cytoskeleton
***VLDLR***	Very-low-density-lipoprotein receptor	Reelin signaling pathway	Abnormal migration. Alzheimer’s disease, temporal lobe epilepsy. ASD

*^a^Bold shows T3-regulated genes that have been found to be abnormally expressed in autistic humans. Other genes found in autistic humans not regulated by T3 at the transcriptional level have not been included*.

Heterotopic cells in the external granular layer of the cerebellar cortex have also been observed (212), as well as in *Mct8*^−/y^ mice (213). The stunted migration of cerebellar cells found in previous studies (6, 214, 215) and in TRα1 mutant mice (212, 213) suggests that thyroid hormones interfere with different mechanisms involved in the migration of cortical and cerebellar neurons. Cortical neurons retain most of their migratory capacity as can be observed either in studies combining BrdU and tracer labeling (205) or using organotypic cultures (Figure 2D) (159). In the latter, it was found that cells from transient hypothyroid medial ganglionic eminence explants migrate as well as cells from control explants when they were placed on normal host cortex; and reversely, both control and transient hypothyroid median ganglionic eminence cells showed altered latero-medial migration when placed on transient hypothyroid host cortex, which suggests that in the transient hypothyroid cortex the expression of chemo-attractive/-repulsive/-stop signals and/or of their receptors [see review in Ref. (126)] is altered. In fact, some of them, such as Slit1, Slit2, and Sema3B, are regulated by thyroid hormones [Ref. (44); Table 2].

### Abnormal cortical cytoarchitecture and connectivity

Blurred neocortical layering can be assessed in the rodent somatosensory barrel cortex owing to the characteristic cytoarchitecture of layer IV (192, 216). The parvalbumin immunostaining pattern in hypothyroid rats is severely altered in the neocortex (211, 217, 218) (Figure 2E) and hippocampus (217, 219). Interestingly, parvalbumin positive neurons (i.e., GABAergic chandelier and basket neurons that migrate tangentially from the medial ganglionic eminence) also exhibit altered tangential migration in the transient hypothyroxinemic cortex (159). The decreased chandelier and basket parvalbumin immunoreactive terminals in the neocortex (211, 217) and hippocampus (217) will affect the inhibitory control of glutamatergic neurons (220) and might explain the high incidence of audiogenic seizures reported in hypothyroid rats (221) and in the pups of mild and transient hypothyroxinemic pregnant rats (158) (Figure 2F).

Early postnatal hypothyroidism affects the growth of dendrites in both the cerebral (193, 222) and cerebellar (223) cortices. Qualitative and quantitative ultrastructural studies of the cerebellar molecular layer in rats show that the retardation in synaptogenesis between Purkinje cell dendritic spines and parallel fibers was associated to hypoplasia of Purkinje cell dendrites and to the retarded development of parallel fibers (223). In the neocortex, it has been found that β-catenin is downexpressed in the dentate gyrus of postnatal hypothyroid rats (165) and the Wnt/β-catenin signaling plays a crucial role for the growth and branching of dendrites (224). Developmental hypothyroidism affects maturation of commissural axons (225–228). In adult hypothyroid rats, the number of myelinated axons was 76 and 66%, respectively, in both the anterior commissure and the corpus callosum compared to controls (228); also, the maturation of cytoskeletal components was altered (226, 229) and the growth of axon caliber was arrested (225, 228). Development and maturation of oligodendrocytes in the forebrain commissures of hypothyroid rats may also be affected. In fact, cortical expression of myelin-associated glycoprotein, proteolipid protein, and myelin basic protein in oligodendrocytes is strongly reduced (230).

Callosal-projecting neurons were found mostly in infra-granular layers of the auditory cortex of developing (205) and adult hypothyroid rats (216). In addition to altered radial distribution, the total number of callosal neurons was increased in auditory (216) and visual (226) cortices, and in cortical projecting neurons such as in the occipito-spinal connections (231), revealing maintenance of exuberant projections in hypothyroid rats. Interestingly, in the hypothyroid MMI model, the heterotopic white matter neurons, in particular, the early BrdU-labeled ones normally destined for the subplate, could provide a target to the transient callosal axons as they might in normal development (134, 232).

### Delayed cortical maturation

**Figure 3:**
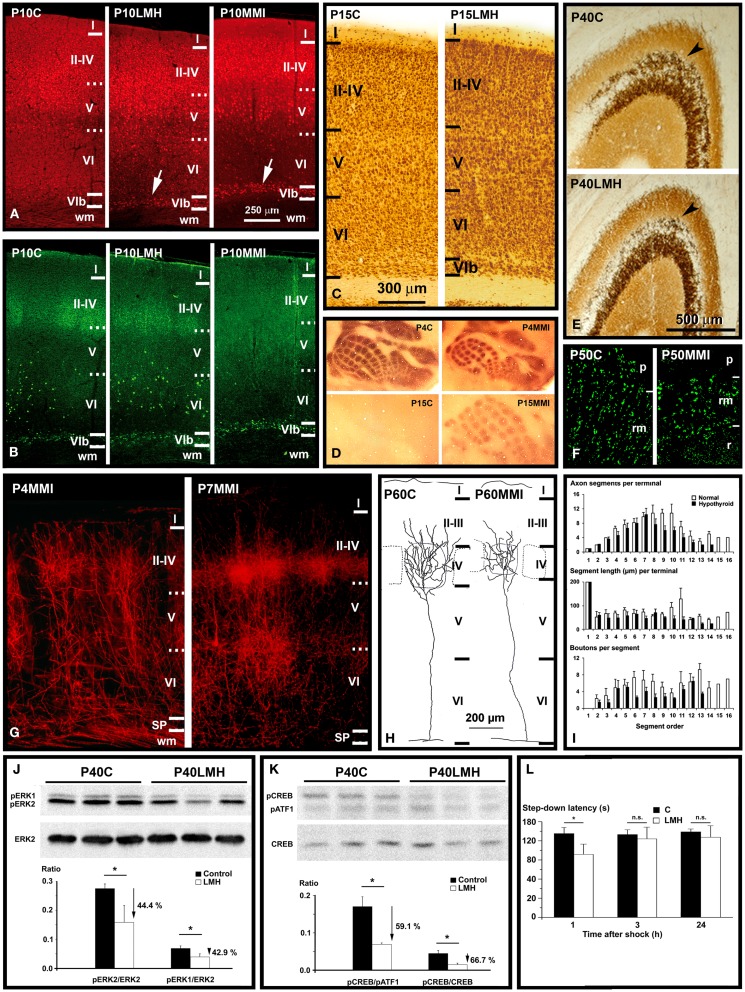
**Thyroid hormones affect connectivity, maturation, and function of the cerebral cortex**. **(A,B)** Collages from confocal photo- micrographs (taken with the ×20 objective) showing double immunolabeling for Camk4 [red; **(A)**] and Nurr1 [green; **(B)**] in the parietal cortex of P10 control (C), LMH (pups born to dams thyroidectomized at E16), and MMI (MMI treatment starting at E10) pups. Most of Camk4-immunoreactive neurons were located in layers II–IV and upper layer V. Numerous Camk4-immunoreactive neurons can be seen in layer VIb of P10LMH and P10MMI pups (arrows) compared with P10C pups. At P10, about 60% of Camk4-immunoreactive neurons of layer VIb are also Nurr1-immunoreactive. **(C)** Photomicrographs of coronal sections of the parietal cortex showing NeuN-immunoreactive neurons in C and LMH pups at P15. At P15, the border between the subplate and adjacent layer VI is more clear-cut in LMH than in C pups, showing that a remaining subplate is still present. **(D)** Photomicrographs of flattened neocortex tangential sections showing 5-HT immunostaining in the posteromedial barrel subfield of the parietal cortex of C and MMI rats at P4 and P15. At P4, heavily immunostained barrels can be seen. Decay of 5-HT labeling occurs by P11 in C and by P16 in MMI rats. Note that in P15C rats, no barrels were immunostained, whereas in P15MMI rats, they are still immunopositive. **(E)** Photomicrographs of coronal sections of the area CA3 of the hippocampus showing the Zn-labeling of mossy fibers in C and LMH pups at P40. Note the heavier labeling of mossy fibers in the stratum oriens (arrowhead) of CA3 in C compared with LMH pups. **(F)** Confocal deconvoluted images of vesicular glutamate transporter type 1 (VGluT1, which labels excitatory buttons) of the stratum radiatum (rm) in CA3 in C and MMI rats at P50, labeling mossy fiber buttons. Note the decreased density of immunoreactive buttons in P50MMI pups compared to controls. **(G)** Photomicrographs of coronal sections from P4 and P7 MMI rats that had the lipophilic carbocyanine DiI tracer (1,1′-dioctadecyl-3,3,3′,3′- tetramethylindocarbocyanine perchlorate) implanted in the ventro-basal thalamic nucleus. At P4, DiI-labeled thalamic afferents enter the somatosensory cortex, and form clusters in layer IV. At P7, collaterals in layer IV form more dense clusters than at P4. These images show that hypothyroid thalamic axons reach their somatosensory target areas as in normal rats. **(H)** Coronal views of thalamocortical terminal arbors in layer IV in the posteromedial barrel subfield of C and MMI rats at P60. The barrel limit is marked with dashed lines. Note that in MMI rats terminal arbors have shorter and tortuous branches. **(I)** Histograms representing mean values for the number of axon segments per terminal (upper), segment length (middle), and buttons per segment (bottom) in C (white bars) and MMI (black bars) rats for each segment order. Note that in general MMI mean values are lower. **(J,K)** Western blots obtained from the hippocampus of C and LMH pups at P40, immunolabeled for ERK2, pERK1, and pERK2 **(J)** and pATF1, pCREB, and CREB **(K)**. Histograms showing that the pERK1/ERK2 and pERK2/ERK2 **(J)** and pCREB/pATF1 and pCREB/CREB **(K)** ratios are reduced by 44.4, 42.9, 59.1, and 66.7%, respectively, in LMH compared with C pups. **(L)** Histogram showing step-down latencies in seconds at 1, 3, and 24 h after the initial foot shock in C and LMH pups at P39. Pups from LMH dams show 24.9% reduction in the step-down latency at 1 h after the foot shock. **(J–L)** Error bars represent ± SD; n.s., no significant differences; *P < 0.001 for LMH compared with C group. **(A,B)** Modified from Navarro et al. (239). **(C,E,J,K,L)** Modified from Berbel et al. (169). **(D,G,H,I)** Modified from Ausó et al. (246).

There is a strong evidence that subplate neurons play an important role in thalamocortical axon path finding (233, 234). Subplate neurons may fire action potentials (235) and they are necessary for the establishment of ocular dominance and orientation columns (236) and for the maturation of inhibitory circuits in layer IV (237). The dynamic integration of subplate neurons into the rodent neocortex during postnatal development may play a key role in establishing the cytoarchitectonic pattern in layer IV and to refine layer IV circuitry (238). Recent studies have shown that subplate neurons remain expressing Camk4 in adult hypothyroid rats, while in normal rats, Camk4 is not longer expressed in subplate neurons by P10 [Ref. (239); Figures 3A–C]. Subplate and white matter abnormalities have been related to the pathogenesis of various brain developmental disorders other than ASD, such as periventricular leukomalacia, schizophrenia, and cerebral palsy (240–244). A recent study shows the crucial importance of the identification of subplate cell subpopulations, which may have very different roles in various pathologies such as ASD and schizophrenia (245).

Serotonin (5-HT) immunostaining is a good transient marker for thalamic afferents in the visual, auditory, and somatosensory areas of rats during the first postnatal days. In the barrel cortex of hypothyroid rats, immunolabeling persisted for 5 days until P16–17 [Ref. (246); Figure 3D]. A similar protracted expression of 5-HT transporter (5-HTT) occurred in the ventro-basal thalamic nucleus and cerebral cortex (246). Reduced 5-HT levels during barrel formation delay the differentiation of layers II, III (247, 248) and reduce the tangential extent of thalamocortical arbors within barrels (249). Thus, prolonged 5-HTT expression in the hypothyroid ventro-basal thalamic nucleus should decrease the concentrations of 5-HT in the extracellular space of sensory cortices, affecting their organization and differentiation.

In the barrel cortex of adult hypothyroid rats, the radial distribution of thalamic afferents, anterogradely labeled with dextran–biotin amine and DiI, was reduced compared to normal rats [Ref. (246); Figure 3G]. By single reconstructions of terminal arbors (Figure 3H), these authors showed a reduction of the number of axonal branches reaching layers II–IV, and a 49% reduction in the total length of terminal axon arbors in hypothyroid rats. This arrested growth was also reflected by a 58% reduction in the number of buttons per terminal (Figure 3I). In hypothyroid rats, ramification of the thalamocortical axons would appear to be stalled postnatally, resulting in reduced synaptogenesis as suggested by the reduced number of buttons in thalamocortical axons [Ref. (246); Figure 3I] and in a decreased number of spines along the apical shafts of the hypothyroid pyramidal cells (250). All of the above data show that many target pyramidal cells fail to reach their correct cortical location, not only failing to complete their normal maturation but also that the afferents have arrested growth. In fact, GAP-43 is downregulated, while Sema3A is upregulated in developmentally hypothyroid and hypothyroxinemic pups (251). In agreement, *GAP-43*^−/−^ mice failed to express 5-HTT in the barrel cortex causing a disrupted segregation of thalamic afferents in the barrel cortex. In addition, recent data show that the density of VGluT1-immunoreactive buttons is decreased in layer IV of the parietal cortex of hypothyroid rats (180). These data show that there is an asynchrony in the maturation of thalamocortical afferents and their cortical targets in hypothyroid rats. Cortical cells could be at a stage of maturation that does not allow them to respond to thalamocortical signals, resulting in abnormal communication between thalamic axons and target cells (e.g., by reduced synaptogenesis). Hypothyroidism seems to dissociate stabilization of juvenile axons from maturation, growth in caliber and myelination, processes, which were previously thought to be necessarily linked (134, 232, 252).

Abnormal patterns of connectivity have been also found in the hippocampus of developmentally hypothyroid rats (193). These authors found in the hippocampus of pups born to hypothyroid dams that CA pyramidal neurons developed atrophic apical (15% shorter) and fewer number of ramifications (about 31 and 36% less in dentate gyrus and CA, respectively). Blurred layering and heterotopic neurons were also found in the hippocampus of pups born to hypothyroxinemic pregnant rats [Ref. (158, 164); Figure 2B]. Decreased mossy fiber zinc density (33–45% reduction) was found in perinatal hypothyroid rats after PTU treatment from E18 to P31 (253) and in postnatal hypothyroid rats (254). P40 pups born to late hypothyroid dams (thyroidectomized by E16; LMH pups), showed a 41.5% decrease in the Zn-positive area in the stratum oriens, in parallel to down expression of the Zn transporter-3 (ZnT-3; Figure 3E) and reduced density of VGluT1-immunoreactive buttons (Figure 3F). In addition, pCreb/pATF1, pCreb/Creb, pErk1/Erk2, and pErk2/Erk2 ratios in the hippocampus decreased in LMH pups (59.1, 66.7, 44.4, and 42.9%, respectively) [Ref. (169); Figures 3J,K]. Recently, the hippocampus of developmentally hypothyroid pups showed altered VGluT1/VGAT immunoreactivity (180). Although Camk4/Creb pathway plays a fundamental role in neurites growth and establishment of synapses, other genes are involved in the development of hippocampal connections. It has been found that the T3-regulated BDNF is involved in the regulation of the translational expression of VGluT1 in cultured hippocampal neurons (255, 256). Interestingly, BDNF was also found involved in the activation of Erk1/2 signaling pathway (188), which affects not only the differentiation of hippocampal neurons but also almost all aspects of corticogenesis (Tables 3–6). However, the inactivation of other pathways such as the Gsk3β/CRMP2 pathway will result in delayed axonal growth (251, 257). Decreased p-Gsk3β labeling and increased labeling of its inactivated p-CRMP2 target protein was seen in the developmentally hypothyroid and hypothyroxinemic rat hippocampus (251).

**Table 3:** **Significant T3-regulated genes at the transcriptional level found in the cerebral cortex of rodents, involved in neurite growth, guidance, branching, and maturation: relationship with ASD**.

Symbol^a^	Protein	Process	Alteration/disease
***ANK3***	Ankyrin-3	Cytosol protein that interacts with voltage-gated sodium channels and cytoskeletal proteins	Abnormal clustering of voltage-gated sodium channels at the axon hillock and node of Ranvier abnormal action potential firing. ASD
***ARX***	Aristaless-related homeobox	Transcription factor	X-linked intellectual disability, epilepsy, lissencephaly, agenesis of the corpus callosum. ASD
***BDNF***	Brain-derived neurotrophic factor	Extracellular signal	Abnormal synaptic structure, function, and plasticity. Fragile X syndrome. ASD
***CAMK4***	Calcium/calmodulin-dependent protein kinase type IV	CREB phosphorylation signaling pathway	ASD
*CHN1*	Chimerin 1 (GTPase-activating protein)	Signal transduction	Abnormal axon pruning
***CNTN4***	Contactin-4	Cell adhesion molecule	Abnormal connectivity in the developing nervous system. ASD
***CREB1***	cAMP-responsive element binding protein 1	Transcription factor	Altered development. ASD
***CREM***	cAMP-responsive element modulator	Transcription factor modulating CREB	Altered development. ASD
***FLT1***	Vascular endothelial growth factor receptor 1	Protein kinase. Signal transduction	Abnormal control of cell proliferation and differentiation. ASD
***FN1***	Fibronectin	Extracellular matrix protein	Abnormal cell adhesion, growth, migration, and differentiation. ASD
*HAP1*	Huntingtin-associated protein 1	Interacts with huntingtin and cytoskeletal proteins	Abnormal vesicular trafficking and organelle transport
*KLF9*	Kruppel-like factor 9	Transcription factor	Altered development of neurons
***MAPK1***	Mitogen-activated protein kinase 1 (ERK2)	CREB phosphorylation signaling pathway	ASD
***NEFH***, ***NEFM***, ***NEFL***	Neurofilament protein (heavy, medium, and light chains)	Intermediate filaments	Abnormal neuronal cytoskeleton. ASD
***NOS1***	Nitric oxide synthase 1	Neurotransmitter, signaling pathway	Abnormal signaling pathway. Neuroglial inflammation. ASD
*PLXNA2,3*	Plexin-A2	Semaphorin co-receptor	Abnormal axon guidance. Schizophrenia, anxiety
*SEMA3B*	Semaphorin-3B	Signal transduction	Abnormal axon guidance
*SLIT1*, *SLIT2*	Slit homolog 1 and 2 proteins	Extracellular matrix protein. Chemorepulsive signal	Abnormal axon guidance. Abnormal angiogenesis
*TGFB2*	Transforming growth factor-β 2	Extracellular signaling protein	Abnormal regulation of proliferation and differentiation of hippocampal granule neurons

*^a^Bold shows T3-regulated genes that have been found to be abnormally expressed in autistic humans. Other genes found in autistic humans not regulated by T3 at the transcriptional level have not been included*.

Altered postnatal synaptogenesis has been observed in the molecular layer of the cerebellar cortex of hypo- and hyperthyroid rats (214). Neurotrophins BDNF and NT3 are downexpressed in postnatal PTU treated rats, resulting in atrophy of Purkinje cell dendrites and in a decreased number of synapses (258) and BDNF is also downexpressed in the hippocampus of developing hypothyroid rats (256). However, neurotrophins might have a dual role in developing and adult hypothyroid rats, because, BDNF is highly expressed in layers II, III, and V of the neocortex and in all hippocampal areas of adult hypothyroid rats (259). These authors observed an increased number of apoptotic neurons and astrocytes in the adult hypothyroid cortex, and they suggested that the increase of BDNF in the hypothyroid adult neocortex and hippocampus might have a protective role against cellular stress associated to degenerative processes. Interestingly, in young and adult autistic human cerebellar samples (ages ranging from 7.5 to 34 years) a 40.3% increase in NT3 expression was found (260). Other T3-regulated genes, involved in synaptogenesis, plasticity, and neurotransmission, have been found [Ref. (23, 44); Tables 4 and 5]. The T3-regulated *ANXA6* gene codes for the calcium-binding protein annexin 6 that may play a structural role in facilitating membrane association at the cisternal organelle level and/or in stabilizing IP3R1 microdomains in the axonal initial segment of hippocampal neurons (261). *NR4A1* codes for the transcription factor Nurr77 that mediates in mechanisms of long-term synaptic plasticity in the hippocampus and consequently, in the consolidation of long-term hippocampus-dependent memory (262). *PACSIN2* codes for protein kinase C and casein kinase substrate in neurons protein 2, which interacts with dynamin and synapsin, which affect the recruitment of synaptic vesicles (263), while *TGB2* codes for transforming growth factor-beta 2, which is involved in the differentiation of granule neurons of the dentate gyrus (188).

**Table 4:** **Significant T3-regulated genes at the transcriptional level found in the cerebral cortex of rodents, involved in synaptogenesis and plasticity: relationship with ASD**.

Symbol^a^	Protein	Process	Alteration/disease
*ANXA6*	Annexin A6	Calcium-binding protein	Abnormal vesicle aggregation and fusion in the hippocampal neuron’s axon initial segment
***ATP2B2***	Ca(^2+^)-ATPase	Plasma membrane calcium-ATPase	Abnormal translocation of calcium to the endoplasmic reticulum in hippocampal neurons. ASD
***BDNF***	Brain-derived neurotrophic factor	Synaptic structure, function, and plasticity. fragile X syndrome autism	Abnormal synaptic structure, function, and plasticity. Fragile X syndrome. ASD
***CAMK4***	Calcium/calmodulin-dependent protein kinase type IV	CREB phosphorylation signaling pathway	ASD
***CNTN4***	Contactin-4	Cell adhesion molecule	Abnormal connectivity in the developing nervous system. ASD
***CREB1***	cAMP-responsive element binding protein 1	Transcription factor	Altered development. ASD
***CREM***	cAMP-responsive element modulator	Transcription factor modulating CREB	Altered development. ASD
*EXOC7*	Exocyst complex component 7	Rho3 signaling	Abnormal cell polarity, regulation of actin polarity and transport of exocytic vesicles
*HAP1*	Huntingtin-associated protein 1	Interacts with huntingtin and cytoskeletal proteins	Abnormal vesicular trafficking and organelle transport
*HRH3*	Histamine H3 receptors	Signal transduction	Abnormal presynaptic inhibition of neurotransmitter release
***MAPK1***	Mitogen-activated protein kinase 1 (ERK2)	CREB phosphorylation signaling pathway	ASD
*NR4A1*	Nuclear receptor related 1 protein (NURR77)	Transcription factor	Abnormal synaptic plasticity in the hippocampus. Altered long-term potentiation. Schizophrenia
***NRGN***	Neurogranin	Calmodulin-binding protein. Component of postsynaptic density	Abnormal synaptic plasticity and long-term potentiation. Schizophrenia. ASD
***PAFAH1B1***	Platelet-activating factor acetylhydrolase IB subunit α (Lis1)	Interacts with dynein and VLDLR	Lissencephaly. ASD
*PICALM*	Phosphatidylinositol binding clathrin assembly protein	Coated vesicles	Abnormal coated vesicles. Alzheimer’s disease
*SLIT1*, *SLIT2*	Slit homolog 1 and 2 proteins	Extracellular matrix protein. Chemorepulsive signal	Abnormal axon guidance. Abnormal angiogenesis
*SNAP23*	Synaptosomal-associated protein 23	SNARE associated protein	Abnormal exocitosis
*SNX16*	Sorting nexin 16	Membrane associated protein	Protein sorting
*SQSTM1*	Sequestosome-1	Ubiquitin binding protein	Abnormal regulation of the nuclear factor kappa-B (NF-κB) signaling pathway
*SYT2*	Synaptotagmin-2	Synaptic vesicles docking	Abnormal exocitosis
*SYTL5*	Synaptotagmin-like protein 5	Synaptic vesicles docking. Marker for parvalbumin immunoreactive buttons	Abnormal exocitosis
*TGFB2*	Transforming growth factor-β 2	Extracellular signaling protein	Abnormal regulation of proliferation and differentiation of hippocampal granule neurons
VAMP4	Vesicle-associated membrane protein 4 (synaptobrevin)	Synaptic vesicles docking	Abnormal exocitosis

*^a^Bold shows T3-regulated genes that have been found to be abnormally expressed in autistic humans. Other genes found in autistic humans not regulated by T3 at the transcriptional level have not been included*.

**Table 5:** **Significant T3-regulated genes at the transcriptional level found in the cerebral cortex of rodents, involved in neurotransmission: relationship with ASD**.

Symbol^a^	Protein	Process	Alteration/disease
*ADCYAP1R1*	Adenylate cyclase-activating polypeptide receptor (PAC1)	Signaling pathway	Decreased second messenger
*CACNG8*	Calcium channel, voltage-dependent, γ subunit 8	Transmembrane AMPA receptor regulatory protein (TARP)	Altered long-term potentiation
***CAMK4***	Calcium/calmodulin-dependent protein kinase type IV	CREB phosphorylation signaling pathway	ASD
***CREB1***	cAMP-responsive element binding protein 1	Transcription factor	Altered development. ASD
***CREM***	cAMP-responsive element modulator	Transcription factor modulating CREB	Altered development. ASD
*HAP1*	Huntingtin-associated protein 1	Interacts with huntingtin and cytoskeletal proteins	Abnormal vesicular trafficking and organelle transport
***HOMER1***	Homer protein homolog 1	Major component of postsynaptic density	Abnormal synaptic plasticity and long-term potentiation. ASD
*HRH3*	Histamine H3 receptors	Signal transduction	Abnormal presynaptic inhibition of neurotransmitter release
*KCNC1*	Potassium voltage-gated channel subfamily C member 1	Membrane channel	Abnormal repolarization of cortical interneurons
***KCNJ10***	ATP-sensitive inward rectifier potassium channel 10	Membrane channel	Abnormal repolarization. Epilepsy, ataxia, and deafness. ASD
*KCNK2*	Potassium channel subfamily K member 2 (TREK1)	Membrane channel	Abnormal neuroprotection against epilepsy and brain and spinal cord ischemia
*KCNS2*	Potassium voltage-gated channel subfamily S member 2	Membrane channel	Abnormal repolarization
*KCNT2*	Potassium channel subfamily T, member 2	Membrane channel	Abnormal repolarization epilepsy, Alzheimer disease
***MAPK1***	Mitogen-activated protein kinase 1 (ERK2)	CREB phosphorylation signaling pathway	ASD
***NRGN***	Neurogranin	Calmodulin-binding protein. component of postsynaptic density	Abnormal synaptic plasticity and long-term potentiation. Schizophrenia. ASD
***NTS***	Neurotensin	Neuropeptide	Abnormal modulation of dopamine signaling. ASD
*PACSIN2*	Protein kinase C and casein kinase substrate in neurons protein 2	Binding to endocytic proteins	Arrested endocytosis
***PAFAH1B1***	Platelet-activating factor acetylhydrolase IB subunit α (Lis1)	Interacts with dynein and VLDLR	Abnormal signaling. Lissencephaly. ASD
***SLC17A7***	Vesicular glutamate transporter 1 (VGLUT1)	Synaptic vesicle membrane protein	Abnormal neurotransmission neuropsychiatric disorders. ADHD, and schizophrenia. ASD

*^a^Bold shows T3-regulated genes that have been found to be abnormally expressed in autistic humans. Other genes found in autistic humans not regulated by T3 at the transcriptional level have not been included*.

Structural changes in the cerebral cortex result in altered electrophysiology and behavior of thyroid deficient rats. Most of the electrophysiological studies have been performed in the CA1 hippocampal area. Decreased long-term potentiation (LTP) was shown in developing rats with severe and chronic hypothyroidism (166, 264), as well as in adult hypothyroid rats (265). Altered LTP was also observed in pups born to rats treated with MMI from E12 to E15 (266). Recent studies have shown that developmental hypothyroidism decreases the number of bursting CA1 cells, as well as the number of spikes per burst, resulting from altered low-threshold Ca^2+^ current (267). As mentioned above, the growth of axon caliber was arrested in the anterior commissure and the corpus callosum of hypothyroid rats (225, 228), which might be relevant for the signal transmission velocity of commissural axons (268). The behavior of hypothyroid rats related to cerebral cortex alterations are mainly based on tests to measure (i) locomotor functional excitability and seizure susceptibility (158, 218, 221, 269), and (ii) learning, attention, and memory deficits (169, 219, 264, 266, 270, 271) (Figure 3L). Despite the differences between rodent and human cerebral cortices, these studies might be useful to find morphofunctional alterations in hypothyroid rodents that might also occur in neurological and mental disorders associated to hypothyroidism in humans, such as ASD and ADHD. Significant T3-regulated genes, involved in memory and behavior have been found [Ref. (23, 44); Table 6]. Among these, *ADCY8*, *ADRBK2*, and *GRK5* code for proteins associated to G-protein signaling (272–274). The transcription factors DBP, involved in kainite-involved seizures and hippocampal plasticity (275), and NR2A1 were mentioned above.

**Table 6:** **Significant T3-regulated genes at the transcriptional level found in the cerebral cortex of rodents, involved in memory and behavior: relationship with ASD**.

Symbol^a^	Protein	Process	Alteration/disease
*ADCY8*	Adenylate cyclase type 8	G-protein associated enzyme	Abnormal cAMP signaling
ADRBK2	β-adrenergic receptor kinase 2 (GRK3)	G-protein-coupled receptor kinase 3	Abnormal dopamine metabolism. Schizophrenia and bipolar disorder
***CALB1***	Calbindin-D28k	Calcium-binding protein	Abnormal synaptic plasticity and long-term potentiation. ASD
***CAMK4***	Calcium/calmodulin-dependent protein kinase type IV	CREB phosphorylation signaling pathway	ASD
***CREB1***	cAMP-responsive element binding protein 1	Transcription factor	Altered development. ASD
***CREM***	cAMP-responsive element modulator	Transcription factor modulating CREB	Altered development. ASD
*DBP*	D site of albumin promoter (albumin D-box) binding protein	Transcription factor	Abnormal spatial learning and enhanced susceptibility to kainate-induced seizures. Epilepsy, schizophrenia, and bipolar disorder
*GRK5*	G-protein-coupled receptor kinase 5	Signal transduction	Memory impairment. Alzheimer’s disease
***HOMER1***	Homer protein homolog 1	Major component of postsynaptic density	Abnormal synaptic plasticity and long-term potentiation. ASD
***HTR7***	5-HT7 receptor	Neuroreceptor	Abnormal learning and memory. Neuropsychiatric disorders. ASD
***MAPK1***	Mitogen-activated protein kinase 1 (ERK2)	CREB phosphorylation signaling pathway	ASD
***NOS1***	Nitric oxide synthase 1	Neurotransmitter, signaling pathway	Abnormal signaling pathway. ASD
*NR4A1*	Nuclear receptor related 1 protein (NURR77)	Transcription factor	Abnormal synaptic plasticity in the hippocampus. Altered long-term potentiation. Schizophrenia
***NTS***	Neurotensin	Neuropeptide	Abnormal modulation of dopamine signaling. ASD
***PVALB***	Parvalbumin	Calcium-binding protein	Alzheimer’s disease and nervous system disorders. ASD

*^a^Bold shows T3-regulated genes that have been found to be abnormally expressed in autistic humans. Other genes found in autistic humans not regulated by T3 at the transcriptional level have not been included*.

## ASD and Thyroid Hormones during Brain Development

Experimental studies in rodents clearly show that thyroid hormone deficiency results in delayed, temporarily, or permanently suppressed, or abnormal, connections, resulting in behavioral and brain dysfunction (10). Abnormal morphophysiological and behavioral traits established during gestation and early postnatal ages might be maintained throughout life and thereby be a risk factor for the development of behavioral and mental disorders later in life.

Despite differences resulting from age at autopsy and the concomitant effects of seizures and of intellectual disability of variable severity, a substantial body of evidence has accumulated since the 1970s on the fundamental morphological changes affecting the brain of patients with ASD (276, 277). Wegiel et al. (278) reviewed available neuropathological data and concluded that ASD results from dysregulation of the normal mechanisms of neurogenesis and neuronal migration, plus dysplastic changes and defects of neuronal maturation. Thus, the neuropathology of ASD is consistent with a prenatal time of onset. A likely etiological hypothesis posits that ASD may be caused by thyroid hormone deficiencies during cerebral cortex development, either due to a genetic deficiency of the *TRIP8* gene (thyroid receptor interacting protein), which codes for a transcriptional regulator associated with nuclear thyroid hormone receptors (279) or associated to maternal hypothyroidism, which increases fourfold the risk of ASD in the child (3, 63).

The morphological brain changes and the genes found to be transcriptionally or functionally involved in ASD will be briefly reviewed here, both from *post-mortem* data and from *in vivo* imaging, along with a summary of the neurotransmitters affected. Finally, the relevance of thyroid hormones in accepted animal models of ASD is presented. Thyroid hormone deficiency diseases and ASD share common altered gene pathways and comorbid disorders, and epidemiological studies reported a relationship between thyroid hormone deficiency and ASD, although morphofunctional differences between these two conditions exist.

### Brain alterations

Young children with ASD are megalencephalic, with increased brain size and weight (276, 277). Brain imaging data and head circumference studies have shown two phases of early brain growth in ASD pathology: early brain overgrowth during the first postnatal years and arrest of growth during early childhood (280), which might be overlapped with neuronal degeneration in some brain regions by preadolescence and continued into adulthood (280, 281). Increased number of neurons could contribute to increase brain volume. Macroscopically and on imaging, the cerebral cortex in ASD exhibits an abnormal pattern of convolutions (282) involving the orbitofrontal cortex (283) and the temporal lobes with hyperconvoluted hippocampus (276). Counts performed in Nissl stained sections suggests increased density of cells in the frontal cortex (284). However, other factors, besides of the increase in the number of neurons, can contribute to increase brain volume. Increased cerebrospinal fluid volume, and slight reductions of gray and white matter volume in frontal, temporal, and parietal lobes have been reported (285). In addition, recent studies have shown focal brain inflammation (286, 287) and increased gliosis subjacent to neuronal degeneration (281). Changes do not affect the brain uniformly, i.e., the fusiform face area and the limbic system have increased cell packing density and smaller neuronal size involving hippocampus, subiculum, and amygdala, and to a lesser extent the entorhinal cortex, mammillary bodies, and septal nuclei (277), while other areas are normal; for instance, the posteroinferior occipitotemporal gyrus showed no differences in pyramidal neuron number or size in layers III, V, and VI (288). In Brodmann areas 44–45, Jacot-Descombes et al. (289) demonstrated reduced pyramidal neuron size suggesting impairment of neuronal networks relevant to communication and social behaviors. However, owing to the relatively small number of autistic brains studied up to date and the enormous heterogeneity in ASD phenotypes and comorbid diseases, more neuropathological studies will be need for clarification of neuroanatomy of ASD (107, 290).

Despite changes in brain volume in ASD, some anatomical alterations are common with hypothyroid brains. Microscopic examination reveals dysgenesis of the cerebral cortex (276, 277) with increased cortical thickness, abnormal laminar patterns, high density of hippocampal neurons, presence of neurons in the molecular layer, neuronal disorganization, poor differentiation of the gray–white matter boundary, and neuronal heterotopias. Cortical neurons are small, closely packed, lack dendritic arbors, and appear immature; these changes are consistent with an arrest of cerebral maturation (290, 291). Also, the cortical organization is altered with narrower cortical minicolumns (292, 293). The focal cortical dysplasia of ASD appears to result from loss of synchronized radial and tangential migration of glutamatergic and GABAergic neurons, respectively (294). The *CNTNAP2* gene, which codes for contactin associated protein-like 2, is expressed in human frontal areas and has been found to be involved in ASD and language impairment (295, 296). A finding consistent with this view is the demonstration by Kotagiri et al. (297) of cytoarchitectural changes in the ependymal cells of the subventricular zone in ASD, with lower cell density in the septal but not in the striatal zone. A subset of ependymal, astrocyte ribbon, and rostral migratory stream (RMS) cells expressed PCNA, Ki67, PLP, and α-tubulin. In addition, the white matter shows areas of focal increase in the number of heterotopias, reflecting abnormal neuronal migration (278). Using imaging, Gozzi et al. (298) showed that the magnetization transfer ratio of the corpus callosum was significantly higher in children with ASD than in normal controls, indicating abnormal myelination in ASD.

According to a consensus by Fatemi et al. (299), reduction in Purkinje cell and cerebellar granule cell density is consistently observed in ASD (300), along with developmental abnormalities of the inferior olives (301), consistent with abnormal neuronal migration before the 3rd month of gestation. Purkinje cells are decreased in the posterolateral neocerebellar cortex and the archicerebellar cortex (302) with vermis hypoplasia on brain imaging (303, 304). Using MRI tractography in children with ASD, Jeong et al. (305) showed decreased fiber numbers connecting cerebellar cortex to ventral and dorsal dentate nuclei confirming a decrease in connectivity and numbers of Purkinje cells.

### Neurotransmitters in ASD

Perry et al. (306) investigated cholinergic biomarkers in the basal forebrain, frontal cortex, and parietal cortex of children with ASD, mental retardation, and epilepsy and found decreased binding of the α4 nicotinic and the muscarinic M1 receptors (α4 nAChR and m1AChR, respectively). In the cerebellum, Lee et al. (307) found decreased α3 and α4 nAChR binding in granule cells, Purkinje cells, and molecular layers along with increased α7 nAChR binding in the granule cell layer. Blatt et al. (308) found that only the GABAergic system was significantly reduced in the hippocampus in ASD; the serotoninergic, cholinergic, and glutamatergic systems were normal. GABA_A_ and GABA_B_ receptor density in the anterior cingulate cortex and fusiform gyrus is decreased (309, 310). The dysregulation of the GABAergic system pathway includes downregulation of GABA_A_ and GABA_B_ receptors (309–311) and reduction of glutamic acid decarboxylase enzymes (312) and metabotropic glutamate receptor type 5 [mGluR5; (313)]. FMRP and mGluR5 are reduced in cerebellar vermis and frontal cortex in ASD (314, 315). In addition, 5-HT neurotransmission has been found to be deficient in ASD; in particular, Oblak et al. (316) showed decrease in 5-HT_1A_ receptor and 5-HT_2A_ receptor-binding density, as well as in 5-HTT in posterior cingulate cortex and fusiform gyrus. Mutations in the GABA_A_ receptor subunit have been associated with ASD and epilepsy (317).

Two relevant genes in the diagnosis of ASD are *SHANK3* (318) and *GABRB3* (311). SHANK3 is a synaptic scaffolding protein enriched in the postsynaptic density of excitatory synapses, and plays important roles in the formation, maturation, and maintenance of synapses. Several *SHANK3* mutations have been identified in a particular phenotypic group of patients with ASD (318). A study of the Danish Newborn Screening Biobank revealed levels of BDNF in the lower 10th percentile during the neonatal period in children later diagnosed with ASD (319). *SHANK3* mutations may be involved in ASD, cerebellar development, and cerebellar vermis hypoplasia (320). *GABRB3* codes for GABA_A_β3 receptor, and is downexpressed in brains of autistic children, particularly in the cerebellum (311).

### Thyroid-related genes involved in ASD

Recently, Betancur (321) concluded that despite the more than 100 genetic and genomic disorders associated with ASD, we still lack a clear understanding of its pathogenesis. In 2007, Castermans et al. (279) identified in a subject with ASD a *de novo* chromosomal anomaly on chromosome 10q21.3 that disrupted the *TRIP8* gene and the nearby *REEP3* gene that codes for receptor expression-enhancing protein 3, which is a microtubule associated protein sequestering the endoplasmic reticulum away from chromosomes during mitosis. The authors concluded that *TRIP8* codes for a protein predicted to be a transcriptional regulator associated with nuclear thyroid hormone receptors but noted that, “no link between thyroid gland and ASD has been reported so far.”

We summarize in Tables 1–6 a list of relevant genes that have been found to be T3-regulated at the transcriptional level in the rodents cerebral cortex (149, 322), and their human homolog genes (marked in bold) that have been found mutated in ASD patients. The list is far to be exhaustive and, most probably, the overlapping between T3-regulated and ASD-mutated (T3/ASD) genes will increase in the near future. Relevant are Creb/Crem transcription factors that are involved in all critical events of corticogenesis, and Camk4 and Erk1/2 kinases that participate in the Camk4/Creb/Crem and Erk1/2/Creb/Crem signaling pathways (44, 183, 323–325).

As mentioned earlier, critical events at the beginning of corticogenesis are cell division and differentiation of neuroblasts to become young migrating neurons, and the migration of young neurons to their final destinations. T3/ASD genes involved in cell division and differentiation (Table 1) are *CTNNB1* codes for β-catenin that is involved in the transition of epithelial-to-mesenchymal transition (symmetrical-to-asymmetrical divisions; see above) and in the astrocytes’ differentiation (326); *DYRK1A* codes for dual specificity tyrosine-phosphorylation-regulated kinase 1A, which is a regulator of brain growth (196); *GNB1L* code for a 6 WD40 repeats-containing protein most likely involved in cell cycle regulation (327); and *FLT1* that codes for vascular endothelial growth factor receptor 1, a tyrosine kinase involved in the control of cell proliferation and differentiation in angiogenesis and neurogenesis; *FLT1* has been found reduced in severe autism (328). T3/ASD genes involved in cytoskeleton organization and cell migration (Table 2) are *GNAS* that codes for G-protein α subunit (Gs-α) (329); *FN1* that codes for fibronectin, an extracellular matrix protein involved in cell adhesion and migration, found increased in serum of children with autism (330); *SERPINH1* that codes for heat shock protein 47 that binds collagen and was found abnormally expressed in the temporal cortex of ASD patients (331); and *NEFH*, *NEFM*, and *NEFL* code for neurofilament subunits and has been found altered in the frontal cortex neurons in children with autism (332). The genes involved in the reelin signaling pathway include *RELN* (reelin), *DAB1* (disabled-1), *VLDLR* (very-low-density-lipoprotein receptor) (331–335), and *PAFAH1B1* (platelet-activating factor acetylhydrolase IB subunit α; Lis1) that interacts with dimein and VLDLR. Fatemi et al. (312) reported decreased blood levels of reelin in children with ASD. Also, using post-mortem material from superior frontal, parietal, and cerebellar cortex from autistic brains and matched controls, significant reductions in reelin protein, reelin mRNA, and dab1 mRNA along with elevations in VLDLR mRNA in frontal and cerebellar cortex, indicative of impairments in the reelin/dab1 signaling pathway in ASD were observed (336). Genetic susceptibility polymorphisms of the *RELN* gene have been described in ASD (337–340), although other studies have been negative (341–344). A recent meta-analysis by Wang et al. (345) revealed that the RELN variant rs362691, rather than rs736707 or the GGC repeat variant, might contribute significantly to ASD risk.

The T3/ASD genes involved in neurite development and maturation (Table 3) are *ANK3* that codes for ankyrin-3, which participates in the recruitment of voltage-gated sodium channels at the axon hillock and node of Ranvier (346); *ARX* that codes for the transcription factor Aristaless-Related Homeobox, associated to several neurological and psychiatric disorders, including ASD (347, 348); BDNF has high-affinity for TrkB receptor and is involved in neurite development, neuronal plasticity, LTP, and apoptosis of CNS neurons (348, 349); *CNTN4* codes for contactin-4, an Ig-cell adhesion molecule involved in the development and plasticity of neuronal circuits (350); NOS1 codes for nitric oxide synthase 1 that is involved in glutamate-mediated neurotransmission and toxicity (351); *FLT1*, *FN1*, and *NEFs* were mentioned above. T3/ASD genes involved in synaptogenesis and plasticity (Table 4) are *ATP2B2* that codes for plasma membrane calcium-ATPase, involved in the translocation of calcium to the endoplasmic reticulum (352); *NRGN* that codes for neurogranin, involved in synaptic plasticity and LTP (353); *BDNF*, *CNTN4*, and *PAFAH1B1* mentioned above.

The T3/ASD genes involved in neurotransmission (Table 5) are *HOMER1* that codes for homer protein homolog 1, is a major component of postsynaptic density involved in metabotropic glutamate receptor signaling (354); *KCNJ10* that codes for ATP-sensitive inward rectifier potassium channel 10, involved in axonal membrane repolarization (355); *NTS* that codes for neurotensin is involved in modulation of dopamine signaling and focal brain inflammation, and was found increased in serum of ASD children (286); *SLC17A7* codes for vesicular glutamate transporter 1 (VGluT1), and is involved in glutamatergic transmission (333); *NRGN* and *PAFAH1B1* were mentioned above.

The T3/ASD genes involved in memory and behavior (Table 6) are *CALB1* and *PVALB* that encode calbindin-D28k and parvalbumin, respectively, are involved in GABAergic transmission (332); *HTR7* that codes 5-HT7 receptor is involved in serotonin signal transduction (333, 356); *HOMER1*, *NOS1*, and *NTS* were mentioned above.

### Animal models of ASD

A number of animal models of ASD are the result of insertion/deletion of different ASD-related genes and exposure to environmental factors [reviewed by Gadad et al. and Provenzano et al. (357, 358)]. Sadamatsu et al. (359) proposed the rat with mild and transient neonatal hypothyroidism as a novel model for ASD. Other models include the repetitive behavior observed in C58/J, C57BL/6J, and Grin1 knockdown mice (360). The homeobox-containing transcription factor engrailed-2 (En2) is involved in patterning and neuronal differentiation; Sgadò et al. (361, 362) showed that adult *En2*^−/−^ mice exhibit reduced brain interneuron expression of GABAergic marker mRNAs, and reduction in parvalbumin, somatostatin, and neuropeptide Y in the cerebellum and cerebral cortex (including hippocampus). The genetically inbred *BTBR T*^+^*Itpr3*^tf/J^ mouse model of ASD exhibits social impairment and stereotypic behavior suggestive of mTOR overactivation (363). The BTBR model shows extensive anatomical abnormalities in the white matter of the corpus callosum and the hippocampal commissure (364). Uchino and Waga (365) identified novel *SHANK3* transcripts whose transcription started at the vicinity of the CpG-island 2 in the mouse brain and developed the Shank3 mutant mice that exhibit autistic-like behaviors. Waga et al. (366) identified two different amino-terminus truncated Shank3 transcripts, Shank3c-3 and Shank3c-4, expressed from the intron 10 of the Shank3 gene, and suggested the epigenetic regulation of the expression of these transcripts via methyl CpG-binding protein 2 (MeCP2). Interestingly, MeCP2 mediates activity-dependent regulation of synaptic strength during the process of circuit formation and prevents uncontrolled recurrent excitation that may result in a pathophysiological increase of neuronal excitability, aberrant network activity, and seizures, which are common Rett patients (182).

The valproic acid model of ASD has become widely used (367–371). However, it is not widely known that valproic acid at the usual therapeutic doses used for the treatment of epilepsy has anti-thyroid effects (372) and induces hearing loss in patients (373).

## Conclusion

Thyroid hormones exert both genomic and non-genomic actions in many tissues, organs, and systems over the course of a lifetime. In particular, they are crucial during early neurodevelopment, since key phases of the CNS development depend of the expression of thyroid hormones regulated genes. These genes affect, among other things, proliferation, migration, and maturation of neurons and glial cells, which under certain circumstances can result in abnormal connectivity, and consequently in behavioral dysfunction. Morphofunctional alterations caused during pregnancy and early postnatal are permanent, and thus they are a risk factor for the development of behavioral and mental disorders later in life. The knowledge of how thyroid hormones regulate these phases of development may help to understand altered regulatory mechanisms in neurodevelopmental diseases such as ASD, ADHD, schizophrenia, and epilepsy with cytoarchitectonic alterations similar to those found in hypothyroidism and hypothyroxinemia and vice versa. By combining basic and clinical investigation, new data will be obtained to better understand the basic phases of brain development and the genetic and physiological events underlying some of the human diseases mentioned above. Despite of obvious differences between humans and other mammals in cortical organization and function, animal models might be a useful tool to approach the understanding of common etiological factors in hypothyroidism and ASD since, as the evo-devo tell us, both rodents and humans share homologous gene pathways involved in these diseases.

## Conflict of Interest Statement

The authors declare that the research was conducted in the absence of any commercial or financial relationships that could be construed as a potential conflict of interest.
